# Culturally Competent Gender, Sex, and Sexual Orientation Information Practices and Electronic Health Records: Rapid Review

**DOI:** 10.2196/25467

**Published:** 2021-02-11

**Authors:** Kelly Davison, Roz Queen, Francis Lau, Marcy Antonio

**Affiliations:** 1 University of Victoria Victoria, BC Canada; 2 Canada Health Infoway Toronto, ON Canada; 3 School of Health Information Science University of Victoria Victoria, BC Canada

**Keywords:** sex and gender minorities, gender studies, information practices, electronic health record, health equity, rapid review, LGBTQ issues

## Abstract

**Background:**

Outdated gender, sex, and sexual orientation (GSSO) information practices in health care contribute to health inequities for sexual and gender minorities (SGMs). Governments, statistics agencies, and health care organizations are developing and implementing modernized practices that support health equity for SGMs. Extending our work, we conducted a rapid review of grey literature to explore information practices that support quality health care for SGMs.

**Objective:**

The aim of this rapid review of grey literature was to elucidate modern GSSO information practices from leading agencies for adaptation, adoption, and application by health care providers and organizations seeking to modernize outdated GSSO information practices that contribute to health inequities among SGMs.

**Methods:**

We searched MEDLINE and Google from 2015 to 2020 with terms related to gender, sex, sexual orientation, and electronic health/medical records for English-language grey literature resources including government and nongovernment organization publications, whitepapers, data standards, toolkits, health care organization and health quality practice and policy guides, conference proceedings, unpublished academic work, and statistical papers. Peer-reviewed journal articles were excluded, as were resources irrelevant to information practices. We also screened the reference sections of included articles for additional resources, and canvassed a working group of international topic experts for additional relevant resources. Duplicates were eliminated. ATLAS.ti was used to support analysis. Themes and codes were developed through an iterative process of writing and discussion with the research team.

**Results:**

Twenty-six grey literature resources met the inclusion criteria. The overarching themes that emerged from the literature were the interrelated behaviors, attitudes, and policies that constitute SGM cultural competence as follows: shared language with unambiguous definitions of GSSO concepts; welcoming and inclusive care environments and affirming practices to reduce barriers to access; health care policy that supports competent health care; and adoption of modernized GSSO information practices and electronic health record design requirements that address invisibility in health data.

**Conclusions:**

Health equity for SGMs requires systemic change. Binary representation of sex and gender in electronic health records (EHRs) obfuscates natural and cultural diversity and, in the context of health care, places SGM patients at risk of clinical harm because it leads to clinical assumptions. Agencies and agents in health care need to be equipped with the knowledge and tools needed to cultivate modern attitudes, policies, and practices that enable health equity for SGMs. Adopting small but important changes in the language and terminology used in technical and social health care systems is essential for institutionalizing SGM competency. Modern GSSO information practices depend on and reinforce SGM competency in health care.

## Introduction

### Background

Sexual and gender minorities (SGMs) face health inequities related to social and institutionalized prejudice [1-5]. Discrimination of SGMs should not happen in Canadian health care, where the ability of individuals to freely express their gender identity is a human right [4-6]. Yet, in its current state, each encounter with the health care system is fraught with the risks of emotional, psychological, and even physical harm to SGMs [1,3-5,7]. Recognition of the impact of outdated gender, sex, and sexual orientation (GSSO) information practices is growing [1-3,8,9], but information practices have changed little in recent decades [2], and are built on outdated, poorly defined, and highly constrained conceptualizations of sex, gender, and sexual orientation [1,2]. Current GSSO information practices are largely outdated and are not reflective of the progress that Canadian society has made in terms of being inclusive of SGMs and defending the human rights of SGMs [4,6].

The aim of this rapid review of grey literature was to elucidate GSSO information practices for SGMs from leading agencies for adaptation, adoption, and application to health care providers and organizations seeking to modernize outdated GSSO information practices that contribute to SGM health inequities. This research extends recent work done by our research team that explored peer-reviewed academic literature [1,2]. What is clear from our previous research is that throughout the Canadian eHealth landscape, GSSO data elements lack useful definitions; fundamentally conflate administrative, clinical, biological, and social concepts; and are structured around cisheteronormative binary constructs of sex and/or gender terms and codes [2-5] that are neither affirmative nor inclusive of SGMs and do not include Indigenous GSSO concepts [2].

### Definitions

GSSO information practices include the definition, collection, use, and sharing of GSSO information. The term *definition* refers to the meaning of the concepts used by humans to communicate and the corresponding concepts and codes used to represent this meaning in electronic health records (EHRs). *Collection* and *use* include clinical documentation, coding, and administrative input of health information into EHR systems to support direct clinical care and communication (primary uses) and organizational needs such as analytics and research (secondary uses). *Sharing* includes standardized protocols for the exchange of health information between health care providers and information systems for primary and secondary uses along a connected care continuum such as messaging standards. The term *providers* refers to regulated and unregulated health care staff and professionals (people employed to provide care). Unless specifically mentioned, we use the term EHRs in its broadest sense—health information systems that contain digital collections of a person’s health history and care records.

## Methods

### Review Questions

Our central question for this rapid review of grey literature was “What are modern and emerging GSSO information practices that reduce barriers to access and to delivery of safe and quality health care for SGMs?” The objectives of this review include the discovery, review, synthesis, and description of SGM equity-oriented GSSO information practices in EHRs from leading experts and organizations.

### Study Selection and Synthesis

The rapid review method was chosen because rapid reviews have gained recognition in health care research as a suitable method for fast exploration of rapidly evolving topics [10] and because this work is a natural extension of and complement to our research team’s recent rapid exploration of peer-reviewed academic literature [1] and EHR systems in Canada [2]. This work expands the domain of inquiry to the body of grey literature that houses key institutional, professional, and technical knowledge of safe, quality, and inclusive health care for SGMs.

**Figure 1:**
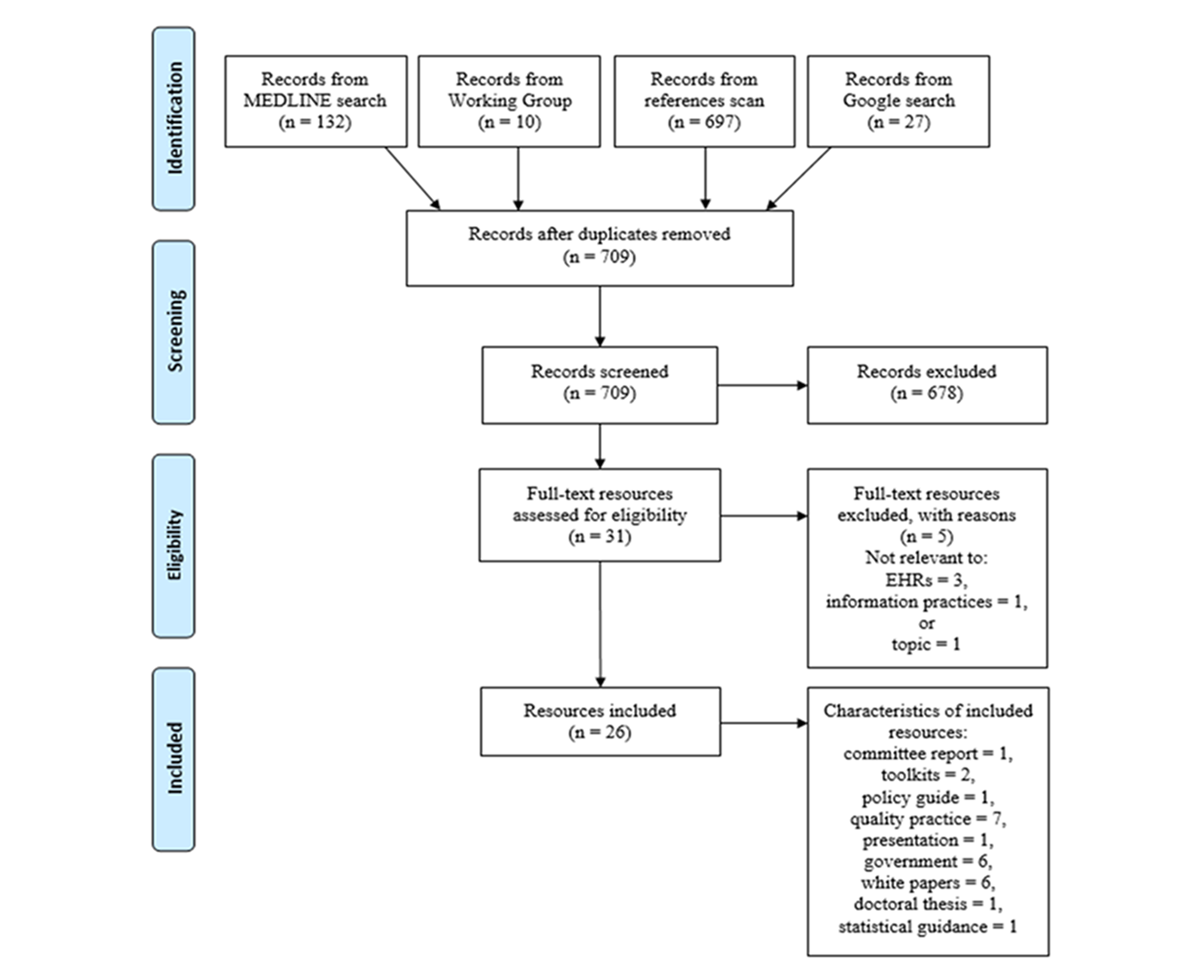
PRISMA (Preferred Reporting Items for Systematic Reviews and Meta-Analyses) flow diagram. EHR: electronic health record.

The search strategy (Multimedia Appendix 1) for this review was developed with the input of a subject matter university librarian and specifies the search terms, including Medical Subject Heading terms, repositories, inclusion and exclusion criteria, and the codebook (Multimedia Appendix 2) developed to guide our analysis. The initial phase of this work involved a search using MEDLINE for English language government reports and documents, white papers, theses, care guidelines, and literature published in a 6-year period between January 2015 and July 2020. The second phase involved a Google search. Search terms for the first two phases included “sex,” “gender,” “sexual orientation,” and “electronic health/medical records.” MEDLINE search results were screened by title, abstract, or description and full-text review for relevance to the topic; peer-reviewed journal papers were excluded. Results from the Google search were screened by hand by reviewing the name of the page and the title of the linked PDF file for relevance. Close screening and selection of GSSO information practice-related resources from the references of each of the included resources were also conducted. Finally, we canvassed an international working group of SGM health experts and organizations for grey literature resources. Duplicates were eliminated. ATLAS.ti was used to support coding and analysis. Researchers met weekly to compare data extraction, ensure consistency of coding, and resolve disagreements through consensus building. Themes were developed through an iterative process of writing and research team discussions. Figure 1 shows a PRISMA (Preferred Reporting Items for Systematic Reviews and Meta-Analyses) [11] flow diagram of the article selection process.

## Results

### Characteristics of Included Articles

The following 26 documents were identified for inclusion: one Canadian parliamentary standing committee on health report [3], two toolkits [4,5], one policy guide [12], seven quality practice documents [7-9,13-16], one presentation on GSSO information best practices [17], six government documents related to changing vital statistics information [18-23], six white papers or data collection standards papers [6,24-28], one doctoral thesis [29], and one statistical guidance paper [30]. Grey literature resources produced by the strategy are summarized in Multimedia Appendix 3.

### Summary of Themes

The primary threads that emerged from the review of grey literature included institutional policies, care practices, social attitudes, and GSSO information practices required to provide safe and quality health care for SGMs. These threads define SGM cultural competence; they naturally and logically constitute the narrative themes that emerged from the literature produced by the organizations addressing SGM barriers to health care through modern GSSO information practices. Cultural competence “…is a set of congruent behaviors, attitudes, and policies that come together in a system, agency, or amongst professionals and enables that system, agency, or those professionals to work effectively in cross-cultural situations” [31]. SGM-competent GSSO information practices are therefore modern GSSO information practices. Modern GSSO information practices emerge from and reinforce the delivery of culturally competent care for SGM. Themes that emerged included (1) essential competency through shared language, terms, and clear definitions of GSSO concepts; (2) the central importance of welcoming and inclusive care environments and inclusive practices for reducing barriers to accessing health care; (3) health care policy that supports competent SGM health care; and (4) modernized GSSO information practices and EHR design requirements that support SGM competence.

### Developing Competency Through Shared Language and Definitions

Gender, sex, and sexual orientation are very different and unambiguous concepts that relate to one another in complex ways for all people [1,4,6,9,16,29]. SGM-competent GSSO information practices are rooted in the language and terminology used to communicate with, for, and about SGMs in health care [4,9]. Standardized definitions and routine use of GSSO data in EHRs support SGM-competent practice by reducing ambiguity, normalizing diversity and inclusion, and affirming SGM human rights. In this section, we provide working definitions of core GSSO concepts.

### Sex

Sex is a complex biological concept that includes anatomy, physiology, genes, and hormones [4,6,15,26]. Biological sex is based on the physical presence of sex organs (physical or phenotypic sex) [15], sex chromosomes (chromosomal or genotypic sex) [15], and hormone levels (hormonal sex) [6].

#### Clinical Sex or Sex for Clinical Use

Clinical sex or sex for clinical use may include some or all the specific concepts of chromosomal sex, hormonal sex, or phenotypic sex, and is applicable to all people. Chromosomal sex does not change, but physical and hormonal sex characteristics can and do change for a wide variety of possible reasons besides hormone therapy (ie, aging and disease) [6]. Sex characteristics may change for a variety of reasons not related to gender transitions, and changes in physical sex characteristics for any patient may therefore warrant procedure-specific adaptations in clinical practice. The screening protocol for imaging [2] may change following a radical hysterectomy related to cancer treatment, as one possible example.

#### Intersex People

Intersex people are born with a range of sex organ characteristics that do not fully conform to traditional assumptions of male or female anatomy [4,15,16]. A binary characterization of sex does not fully represent the natural spectrum of chromosomal, hormonal, and anatomical variation [16]. Labeling intersex people (those people who identify as intersex) with terms, words, or concepts that are not intersex, such as *transsexual*, *undifferentiated*, *unknown*, *ambiguous*, *male*, *female*, and *other*, in their health information may be stigmatizing.

#### Sex Assigned at Birth

When a person is born, the current practice is that they are assigned a sex based on their external anatomy [4,6,15]. The newborn’s sex information is documented in care records and is also submitted to vital statistics (ie, birth certificates). The external genitalia of some people are altered at or around birth via “normalization” procedures [3]. We put the term normalization in quotes because naming such surgeries in this manner may be considered a tangible example of cisgenderism, where binary male and female anatomies are considered normal and anatomies that do not fit within the traditional binary assumptions for anatomy are not normal, with these procedures being undertaken to correct the abnormality. There are growing calls to end these practices unless they are absolutely necessary, and practitioners would ideally wait until the person has appropriately matured and informed consent can be fully supported [3].

### Gender

Gender is a complex human psychological and sociocultural concept that can impact sex characteristics [15,17,26]. Like sex, gender is often erroneously conceptualized and represented as a binary construct (i.e. man/woman), but there is considerably more variation than this in reality [4,6,17]. Gender concepts should not be conflated with sex concepts, but very frequently they are. Gender is both expressed and felt as a part of a person’s identity. As such, the concept of gender and gender-related labels are frequently inappropriately applied to patients by external agents and then used inappropriately in clinical practice.

#### Gender Expression

Gender expression is the way people outwardly display their gender [1,4,17,26,30]. There is no single or correct way to express gender; gender expression is dynamic, situational, and individual. People may express different gender identities at different times and in different situations. Only about half of all transgender people live in their felt gender full-time [6], which means that lived gender (or gender expression) may change across different encounters with health care. Lived gender is the gender they express outwardly the majority of the time [30]; lived gender is a person’s typical gender presentation. It should therefore not be assumed that gender presentation is congruent with lived gender or gender identity.

#### Gender Identity

Gender identity is a person’s inner sense or felt gender [1,4,6,8,17,26,30]. Gender identity is different from lived gender because a person may not feel comfortable living/presenting outwardly in their felt gender.

#### Names, Pronouns, and Honorifics

Names, pronouns, and honorifics are central to a person’s gender identity and are expressions of gender identity for all people [4]. Utilizing chosen names and pronouns is particularly important in the competent care of transgender people because they are affirming of one’s gender [4,13,16,29]. *Legal name* is the name that appears on official government identification documents (ID) and is typically the name that was recorded into government and health records at birth. *Chosen name* or name used is the name that people choose to use in interactions. Chosen name may or may not be reflected in government records [4]. *Pronouns* can be gendered (he/him/his and she/her/hers) or gender neutral (them/they/theirs) [4,5]. *Honorifics* are often gendered and include Mr, Mrs, Ms, Ma’am, and Sir [4,5].

#### Cisgender, Transgender, and Nonbinary Gender Identities

A cisgender person’s felt gender identity matches their sex assigned at birth; a transgender person’s gender identity does not [4,15,26,30]. Some people whose felt gender is not a match with their sex assigned at birth may identify with a binary option as their felt gender [4-6]. People with binary gender identities identify as either a man or a woman. People with nonbinary gender identities do not identify within the man/woman binary [15] and may identify as nonbinary, enby, genderfluid, genderqueer, agender, and bigender [4,5,13,25].

#### Transition

Transition is the term used by transgender people to encompass the complex dynamic social, public, and sometimes medical process of changing one’s gender expression to match their felt gender [4]. Transition can involve changes to chosen names and pronouns used [4,5], clothing and jewelry worn, voice, vocabulary, mannerisms, anatomy, and physiology [4,16]. Transition may also involve changing legal records to reflect lived gender or gender identity, such as changing one’s name [4] or legal gender [5,22]. People who transition may or may not identify using a binary gender term during or following transition.

### Sexual Orientation

Sexual orientation is a term that broadly describes how a person characterizes their emotional and sexual attraction to others [4,12,13,26] and is a complex facet of human sexuality.

Human sexuality is dynamic and multidimensional, and includes a constellation of interrelated concepts such as sexual identity, sexual attraction, and sexual behavior [8,26]. For some people, these concepts align with monogamous heterosexualism. For others, there are important differences. Sexual identity depicts how a person self identifies (eg, lesbian, gay, bisexual, pansexual, and asexual) [4,7,26], and includes both how a person views themselves and what groups they consider themselves to be part of. Sexual attraction describes what gender group or groups one is or is not attracted to [4,7,26]. Sexual behavior refers to the sex acts that one engages in and may be periodic (eg, 1 year) [4,7,26]. Sexual behaviors may occur within one’s sexual and/or romantic partners, but also may occur outside of relationships, in groups, or with oneself [26]. Sexual orientation does not strictly determine sexual behaviors [4], and assumptions about sexual behavior should not be made based on sexual orientation.

Health assessments should include information about sexual behaviors and context for all people, regardless of sexual orientation [8], because behavior and context are important factors in the characterization of risk and in the subsequent formulation of relevant preventative teaching and interventions.

### Two-Spirit

The Indigenous concept of Two-Spirit pre-dates Western colonial frameworks for sexual and gender diversity [8]. Ongoing colonial forces include the structural imposition of belief systems that condemn sexual and gender diversity and enshrine cisheteronormative binary constructs. This fuels transphobia and homophobia, resulting in Indigenous SGMs sometimes being forced from their home communities [5]. The English language term Two-Spirit was adopted by Indigenous SGMs for Indigenous SGMs in 1990 at the third gathering of Gay and Lesbian First Nation People in Winnipeg, Manitoba, Canada; the term Two-Spirit can encompass gender, sex, culture, sexuality, and spiritual identities [3-5,15,32]. Not all Indigenous people who are SGMs are Two-Spirit [5]. Adopting a stance of cultural humility and including Indigenous GSSO concepts is essential for culturally safe and SGM-competent health care for Indigenous people [9]. Work in this space must center around, be led by, and involve earnest partnership with Indigenous Two-Spirit people and engage with ongoing processes of decolonization and anti-Indigenous racism.

### Addressing Barriers With Welcoming Health Care Settings and Affirming Practices

In addition to a shared understanding of language and terminology, enabling safe, quality, and inclusive care for all people requires welcoming care environments [4,5,7]. EHRs that support expanded GSSO concepts, and psychologically safe and meaningful care interactions between patients and health care staff are essential structures that drive and support modern practices [7]. Modern GSSO information practices are free of assumption and stereotype, and meaningfully address psychological safety of care environments, administrative interactions, care interactions, and other barriers to access. Administrative databases, anatomic inventories, and special considerations for the challenges faced by transgender, Indigenous, older, and newcomer SGM people are core foci for health equity.

### Welcoming Care Environments

Health care ought to be affirming and welcoming; a place where patients can feel accepted, safe, and cared for [4,5]. All health care staff are ambassadors of the health care system and should therefore receive SGM-competency training [15]. Affirming health care environments address a key barrier to access for SGMs (pervasive cisheteronormativity) [4,5]. Symbols of diversity and inclusion, such as rainbow flags, affirming images, posters that signal respect and inclusion, and nongendered bathrooms [4,5,8,12,13,15,16,29], send the message that health care is safe and welcoming of diversity. The use of culturally competent language and terms [9,13,15]; careful respect of patient privacy and confidentiality [4,8,30]; and avoidance of assumptions about patient names, pronouns, honorifics, gender identity, sex at birth, and sexuality are essential because assumptions can lead to negative experiences for both patients and staff [16]. Every interaction with health care should not be discounted in terms of its overall importance to ensuring equity. Competencies of services and providers within them are assessed by each patient at each interaction, and decisions about accessing health care or recommending care to others are made. As a logical consequence of experiencing stigma and discrimination, SGMs may not feel comfortable disclosing details about their sexual orientation, sexual behaviors, or gender identity [4,12,29]. Patients should not be made to feel forced to disclose GSSO information.

### Modernized GSSO Information Practices

SGM-competent practices avoid assumptions, misgendering, outing, and deadnaming, protect patient privacy and confidentiality [4], and ensure that current GSSO information is collected and documented into EHR systems to maximize the safety and quality of primary and secondary uses [17]. Modern GSSO information practices are expanded to be inclusive and support affirming care by ensuring that name, gender identity, and pronouns used are meaningfully integrated into technical, social, and organizational layers of health care systems [24]. Routine collection of GSSO information is important for measuring, monitoring, and improving population health [8].

### Patient Registration

Patient registration involves gathering key administrative and demographic information from patients and entering that information into an electronic or paper patient record. Registration should be done in a private nonintrusive manner for all people [7,15,16], and in addition to being staff facilitated, it can be done through transcription of a paper form [7], direct patient entry self-interviews via a patient portal [7,16], a self-serve kiosk [16], or a mobile device [7]. When summoning patients to a registration desk is necessary, calling them by their last name without the use of gendered honorifics, pronouns, or legal names is a useful practice for avoiding negative experiences until preferred names, pronouns, and honorifics are confirmed by the patients and their consent to share that information with the care team is obtained. Patients may be more comfortable sharing GSSO information using self-interviews [12]. The dynamic nature of gender identity should be considered in patient intake and registration workflows, and GSSO information should be confirmed regularly [4,7,8,16]. Consistent and appropriate gender identification and use of appropriate names and correct pronouns for patients are simple yet important actions for providing affirming health care [4,5,8,13] at and beyond registration, and they demonstrate organizational and professional commitment to an inclusive and affirmative health care culture.

### Patient Identification

Governments are beginning to modernize vital statistics to be more SGM inclusive. At the federal level in Canada, citizens who do not identify with binary sex or gender designations can have an “X” printed on their passport, travel document, citizenship certificate, or permanent resident card [4,19,20]. Provinces, including Ontario, British Columbia, and Manitoba, are also aligning their systems to enable more diverse gender or sex designations on government-issued IDs through a similar mechanism [21-23].

Government-issued IDs are considered trusted sources for identifying patients for health care purposes [15]; however, there is a high degree of conflation of the two concepts within jurisdictional information systems [2] and subsequent variation in how administrative sex and gender data are labeled and presented on IDs from jurisdiction to jurisdiction. Administrative sex and/or gender on government IDs may not match with the gender identity (or felt gender), gender expression (or lived gender), or sex assigned at birth; administrative gender may even be labeled as *sex* on government IDs [22]. Use of administrative sex or gender for clinical care can lead to substantial confusion in information systems and clinical care and can increase the risk to patient safety.

A two-step approach that includes sex assigned at birth and gender identity is considered the best practice for collecting gender and sex information by leading organizations [6-8,13,15,24] because it eliminates conflation between administrative and clinical sex and/or gender concepts and reduces conflation-related risks. However, health organizations must respect the patient’s choice to not provide this information, and staff are strongly cautioned to avoid making assumptions about patient identity or applying labels to patients that patients themselves may not identify with (ie, transgender) based on this information. Administrative sex and/or gender should not be labeled or documented as anything but administrative sex and/or gender to avoid conflation with clinical concepts. Nonbinary people may not wish to have gender markers on their documents [21]. Personal health numbers can, and should, be used for positive identification in the case of mismatches in administrative sex and/or gender information.

### Clinical Encounters

Thorough assessment and documentation of medical and surgical history in postregistration encounters can help ensure that opportunities for appropriate screening are not missed [4,13]. Preferred names and pronouns should be collected in the first visit [8]. Staff can positively identify their own names and pronouns when first meeting with a patient to build trust and a sense of safety [4,16]. Providers should ask about sexual orientation, sexual behavior, and gender identity using open-ended questions [8,12], and should confirm names and pronouns used with the patient so that they can correct any incorrect GSSO information that has been erroneously documented in the patient chart and minimize the possibility of it being used in subsequent clinical encounters. If affirming approaches are unsuccessful, GSSO information may be collected as part of a more formal and more focused sexual health assessment [12]. Clinicians who ensure that documentation is complete, accurate, and up to date with current GSSO information reduce the chances for negative experiences for both patients and providers in subsequent care interactions [24].

GSSO information should be collected without the presence of parents for youth aged 13 years or above, but parents should be involved in the conversation for children aged 12 years or below [7].

### Addressing Health Inequities

Gaps in preventative screening for SGMs exist because outdated GSSO information practices and binary administrative sex and/or gender data constructs interfere with appropriate matching of SGM patients’ preventive screening and health care needs [4]. Anatomic inventories enable transparent and assumption-free therapeutic relationships between patients and providers. Safe therapeutic relationships lead to increased opportunities for prudent screening and prevention [1,2,4,5,9].

#### Anatomic Inventories

Anatomic inventories are objective catalogues of anatomical parts that allow assumption-free unambiguous representation of diverse bodies to support appropriate screening, treatment, and referral decisions matched to biological needs [1]. If a person has a particular body part or organ and otherwise meets criteria for screening based on risk factors or symptoms, screening can and should proceed regardless of hormone use [13] or other sex and/or gender characteristics or identities. Anatomic inventories ought to be evaluated as the best practice for all people given that they eliminate unconscious profiling based on sex assigned at birth or administrative gender. Providers are encouraged to maintain a current anatomic/organ inventory in their patients’ charts to guide quality care planning [13].

#### Screening Considerations

SGMs are diverse [16] and face specific barriers to health care and associated health risks [2,4,13] that necessitate differential screening [1,8]. SGMs are at higher risk of depression, suicidal ideation and attempts, substance use, chronic disease, sexually transmitted and blood-borne infections/illnesses, homelessness (especially youth), and food insecurity [3], and should be routinely and competently screened [13]. Long waitlists and overly complex assessments and referral processes, often from clinicians in private practice (eg, psychologists, psychiatrists, and social workers), for gender-affirming surgeries are possible barriers to access [3], and sex designation on government IDs and health documentation may affect billing and eligibility for sex-specific diagnostics or procedures [29].

#### Transgender SGMs

Transgender people are made particularly vulnerable by health care that is not SGM competent. An estimated 30% of transgender people do not engage with emergency services when needed because of the perceived risk of being stigmatized, and half of those who do engage with health care report negative experiences [3]. It is important to note here that gender dysphoria is felt differently by different people regarding different aspects of their bodies, gender role, or assigned sex [5]. Not all transgender people seek intervention or support with transition beyond those necessary to affirm their own gender identity [4,13]. However, limited access to gender-affirming surgeries is associated with high-risk behaviors seeking to address intense gender dysphoria such as self-injection of silicone obtained from illicit sources without medical guidance, supervision, or follow-up [13]. Trans Care BC and Alberta Health Services are health organizations that have both released toolkits to support gender-affirming care practice in Canada [4,5].

#### Indigenous SGMs

Two-Spirit and Indigenous SGMs face discrimination and stigma because of their sexual orientation, their gender identity, and, in some cases, their HIV or hepatitis C virus (HCV) status [3]. Culturally safe and SGM-competent care practice for Two-Spirit is critical for reducing barriers to access and for improving health outcomes for Indigenous SGMs [3-5].

#### Older SGM Adults

Older SGM adults are at risk of social isolation and limited social support due to historic criminalization and ongoing stigma. In the absence of SGM-competent programs along the continuum of care (ie, SGM retirement homes), older SGM adults may face the decision to recloset in order to avoid the trauma of being stigmatized by other patients and staff in unfriendly and unwelcoming care environments [3].

#### Newcomers

SGMs who immigrate face additional barriers that should be considered by clinicians providing care [3,9]. Cultural ways of expressing gender, sex, and sexual orientation in language, behavior, and appearance may vastly differ from practices in mainstream Canada. Furthermore, the intensity of stigma, trauma, and persecution that people may have faced before immigrating is an essential consideration in the care of newcomer SGMs. Cultural competency (ethnic) is central to cultivating health equity for culturally diverse people [14]. Providers should consider the following when providing care for newcomer SGMs:

- The immigration process in Canada can be stressful and costly and can involve psychiatric and other assessments [3]; newcomers may become aware of their HIV or HCV status through the process and should be offered appropriate counselling and support.
- Gender-positive messaging in languages other than official languages can help reduce language as a barrier to accessing health care, and anonymous and confidential access to sexual health services can increase opportunities for screening, prevention, referral, and treatment [3].
- Expanded gender identifiers can enable less stigmatizing immigration processes [19,20].

### SGM-Competent Health Care Policy

In 2017, the *Canadian Human Rights Act* was amended to include gender identity and gender expression as prohibited grounds for discrimination [4,6]. All Canadians have the fundamental right to identify and express gender in a manner that is consistent with their identity [4]. The requirement for mandatory ongoing training for all staff in health care (physicians, nurses, social workers, clerks, administrative staff, and others) is aligned with the objective of improving knowledge and changing attitudes toward SGMs in support of these rights [3,4,27,29]. Health care policies and procedures should be evaluated for diversity, equity, and inclusion.

### Training and Education

Health care providers have the responsibility to ensure respectful, compassionate, and equitable interactions with patients [16]. Since cultural competency requires changes in the attitudes and beliefs of the people who provide health care, both clinical and nonclinical staff require regular SGM-competency training [13,15] and evaluation. Educating staff about modern GSSO information practices is an essential step toward SGM-competent care [4,14,17]. SGM competency training reduces the knowledge gap and increases opportunities for psychologically safe therapeutic conversations between patients and providers. In turn, this can improve access to care [27] by reducing the barrier of stigma and the burden of education that many SGMs feel [3-5,29]. It is not uncommon for SGMs, especially transgender people, to have to educate their providers about their own care [29]. Providers should be receptive to this as a form of patient self-advocacy and engage in their own professional development [14]. Learning about the factors that impact SGM health may mean having to reflect on personal bias [4] and obtaining a nuanced understanding of GSSO.

Staff should be trained in small groups to support psychological safety. Health information technology staff should begin training early [8] because inclusive data structures support affirmative competent clinical practice and organizational culture [7]. Staff from EHR vendors and insurance companies also need to be educated on the health needs of SGMs [7]. Enthusiastic champions should be used to lead culture change in these organizations [8]. Digital solutions for training can be offered and completed remotely and are one option for addressing disparities in urban-rural service offerings [5,8].

### Policies for Discrepancies in GSSO Information

EHRs facilitate instantaneous communication of accurate and inaccurate information among the care team. Policies and procedures for correcting inaccurate GSSO information at registration are required to avoid downstream confusion of terms [15] and risks to patient safety. Inclusion of expanded unambiguous GSSO concepts in EHRs provides appropriate space for GSSO information and is necessary to eliminate health data invisibility and to support SGM-competent care [7]. Where discrepancies exist between labels, codes, and data elements, labels and data dictionaries should be corrected quickly to avoid harm to patients resulting from their use in clinical care.

### Laboratory Policies

Clear organizational support and guidance for GSSO laboratory information practices is required to ensure patient safety [15] because laboratory results frequently inform clinical interventions such as the appropriate dosage of medications. Changes to administrative gender codes by jurisdictions may impact downstream laboratory and EHR systems [15]; implementation requires planning, particularly as it relates to the gender or sex value “X” [4,8]. Incongruencies in sex, gender, and name listed on samples should be confirmed, rather than discarded [16]. Clear labeling on screening samples is required to ensure that samples are processed correctly, despite the possibility of gender marker incongruence between EHRs and laboratory information systems [13,16]. Sex assigned at birth, legal sex, and gender identity data elements provide necessary information for interpretation [15], and incongruence between gender elements and sex at birth can guide safe laboratory interpretation and reporting practices [16] provided that staff have the required competency. Making both male and female reference ranges available to clinicians can improve safety for SGMs [8]. Hormone levels for nonbinary people may intentionally be in between typical male and female levels [13].

### Privacy and Confidentiality

Policies governing privacy and confidentiality are a cornerstone of SGM-competent health care. It is quite logical that SGM patients may be reluctant to share information about themselves, particularly in the context of open waiting rooms or areas where privacy cannot be assured [12] given the risk of stigma-related trauma. Privacy is very important to SGMs, especially transgender people [30] and people who have experienced harm via public acts of being misgendered, outed, or deadnamed in previous health care experiences. Organizations that collect GSSO information must ensure that the privacy and confidentiality of patients is maintained [4,7]. Technical safeguards must be in place to ensure privacy and confidentiality of the EHRs and data within them [12], and patients can be informed about these safeguards. SGM-competent health care, with modern GSSO information practices, addresses risks related to breaches in privacy and confidentiality for SGM patients [4].

#### Information Sharing

EHRs enable the instantaneous sharing of health information. While sharing of limited patient information between providers is sanctioned so long as it is limited to authorized purposes (ie, continuity of care) and follows specific security protocols [9], providers should still obtain consent to document GSSO information in patient charts. Patients can be assured that their health information will be kept confidential [12] according to health privacy legislation [4] and will not be shared unnecessarily.

### Informed Consent

Informed consent is an important patient right and, where applicable, should include reproductive and fertility considerations [13] for the wide range of gender-affirming hormonal therapies and surgical procedures that are available [4]. Transgender people, for example, have the same range of reproductive desires as nontransgender people; reproductive goals for gender-affirming procedures should be considered before they are undertaken. Gonadectomies, for instance, will have clear impacts on fertility that should be discussed [13].

### Insurance Coverage

Costs for medically necessary procedures are a barrier to health care and should not be borne by patients alone. Reimbursement policies should recognize the unique health needs of SGMs [3,7], and insurance companies should support gender-affirming surgeries that are deemed medically necessary [3]. Binary administrative sex and/or gender structures that govern coverage eligibility for certain procedures are commonly cited as problematic barriers to access and to health equity for transgender people [29].

### Conversion Therapy

Conversion therapy is a broad term that encompasses health care practices intended to change an individual’s sexual orientation or gender identity. Conversion therapy is considered to have negative effects on the mental health of SGM patients, and there have been calls to ban such practices in progressive jurisdictions [3].

### Modern GSSO Information Practices and SGM Competency Within EHRs

EHRs support clinical practice and are an extension of clinical culture. Adoption of modern GSSO information practices in EHRs addresses the invisibility of SGMs on user interfaces, in data repositories, in health terminology, in classification and coding schemes, and in messaging and exchange standards. EHRs designed to support modern GSSO information practices improve outcomes by supporting SGM-competent health care.

### Standardized GSSO Data Collection

Data invisibility and institutional erasure of SGMs are increasingly recognized as structural problems contributing to poor outcomes for SGMs [3,6,7,16]. The passive erasure of gender and sexual diversity in EHR code systems has led to inaccurate documentation, unsafe care, invisibility of SGMs in health data [16], and inaccurate information for health care providers, health information professionals, and researchers [7,33]. Addressing the erasure of SGMs in the data means developing and implementing unambiguous data standards as well as methods for analytics and statistics that are inclusive of SGMs. Policies and practices that support routine collection of expanded GSSO information are essential.

### Routine Collection and Use of Standardized Expanded GSSO Concepts

EHRs must include expanded GSSO concepts [7,16,17] or else they risk acting as barriers to quality care [29]. The use of expanded GSSO data elements (beyond the binary) enables culturally competent and clinically safe care and provides a richness of discrete variables for clinical care and analytic uses that does not currently exist in most EHRs [1,6,15-17,29]. Routine standardized collection of GSSO information that includes expanded GSSO concepts is recommended by health care quality organizations as best practice and as a foundational step to understanding and addressing inequities for SGMs [7,14].

### Not Using “Other” as a Code

Use of *other* as a sex or gender code is a common and outdated information practice in EHRs [2] and statistical surveys [18,26]. It is often done with the intent of capturing sex and gender data that are not binary male/female or man/woman, but has the effect of normalizing binary elements and obfuscating the range of possible nonbinary elements. This practice creates ambiguity in code sets and data sets and can be stigmatizing for SGMs, particularly in the context of patient-user interfaces where a person may be forced to “other” themself via a health care artefact. *Please specify* is one option [18] that can provide space for entering correct information where correct options do not exist. However, a full complement of discrete expanded GSSO terms is preferable because discrete elements can support logic-based rules, decisions, algorithms, and artificial intelligence.

### Health Analytics and Statistics

SGM health information facilitates improved knowledge about their health and liberates evidence that can be applied for quality improvement [7,26]. Gender-based analysis is important for statistical comparisons [30]. National [3] and international [30] efforts to address data invisibility for SGMs are now underway by national statistics organizations [6,26]. Due to the small proportion of people with diverse gender identities, special statistical considerations are required to prevent breaches of confidentiality on result maps and to ensure inclusiveness in mathematical and statistical modeling [2,30]. In order to harmonize and strengthen the measurement of health inequalities in Canada, the Canadian Institute for Health Information has proposed Canadian equity stratifiers (standard data elements) that support quality health information for improving outcomes [6].

### Administrative and Legal GSSO Concepts

Administrative or legal GSSO concepts are found in jurisdictional repositories and are used by governments for administrative reasons as minimum data elements collected and used for vital statistics, by insurance agencies for billing, and by health care organizations for patient registration [1,2,15,19-21,23,28]. Administrative GSSO concepts include legal name, administrative gender, administrative sex, and sex assigned at birth, amongst others. Many jurisdictional repositories represent sex and gender concepts as a single data element using a male/female binary supplemented by a category of *other*, *unknown*, or *undifferentiated* [1,2]. The integration of administrative and health care information systems must be done carefully such that administrative and clinical GSSO data elements are appropriately sequestered. Clinical data elements must be clearly labeled and easily differentiated from administrative sex and/or gender data elements in order to support safe and SGM-competent clinical care.

## Discussion

### SGM-Competent GSSO Information Practices as an Equity-Oriented Intervention

Health equity is the absence of unfair differences in access to quality health care or health outcomes [6,16], and is supported by modern GSSO information practices that aim to reduce barriers to access and provision of safe and quality health care to SGMs. Equitable access to quality health care is a right for all Canadians and should be a right for all people including SGMs.

### Beyond the Binary

Binary representation of sex and gender in EHRs obfuscates natural and cultural diversity and, in the context of health care, places SGM patients at risk of clinical harm because it leads to clinical assumptions. Outdated GSSO information practices render SGMs invisible in therapeutic interactions, data sets, and bias analytics, and ensures that researchers have little quality SGM data with which to improve health care for SGMs.

### Clinical Care That Affirms Gender Identity

Knowledge and use of inclusive and gender-affirming language in clinical interactions in the context of welcoming care environments with SGM-competent staff and EHRs that include and display unambiguous GSSO information are all key elements of modern GSSO information practices. Modern GSSO practices are assumption free, unambiguous, and antistigma, and are necessary to support the psychological safety of SGM patients in health care spaces. Modern GSSO information practices support quality therapeutic relationships that, in turn, enable thorough and appropriate screening, care, and referral of SGM patients.

### Moving Forward

Health care system performance may continue to improve as new and innovative technologies are implemented, but if GSSO information practices are not modernized, the health inequities of SGMs are likely to persist or worsen [6]. Further research is needed to improve primary care for transgender people [29], to development specific laboratory guidelines and reference ranges for the gender diverse community [15], and to improve the accuracy of hormone therapy [16]. SGM-inclusive analytics and outcome measures need to be developed, and future research projects should consider using an intersectional lens to gain further insight into structural factors such as institutionalized prejudice faced by SGMs. Anatomic inventories are a core tool that should be evaluated as the best practice for modern GSSO information practices in health care for all people. Both private and public sector health technology regulations ought to include specifications for interoperable SGM-competent system design, including the use of expanded GSSO concepts and definitions in EHRs.

### Institutionalizing SGM Competency

Health equity for SGMs requires systemic change. Agencies and agents in health care need to be equipped with the knowledge and tools needed to cultivate modern attitudes, policies, and practices that enable health equity for SGMs. Adopting small but important changes in the language and terminology used in technical and social health care systems is essential for institutionalizing SGM competency. Modern GSSO information practices depend on and reinforce SGM competency in health care.

### Limitations

The primary limitations of this review include language and timeframe. Only English-language documents published in the past 6 years and indexed within a single database (MEDLINE) were included. The authors are not bilingual. Publication year range was limited for expediency and for recency. Relevant documents from outside of the time frame or published in other languages or databases may have been missed. All included articles were from Western countries and cultures, limiting generalizability to other settings and creating a bias for Western ideals and strategies. Efforts to mitigate these limitations included seeking resources from an international group of experts and stakeholders that represented non-Western countries and non–English-speaking persons.

## Appendix

Multimedia Appendix 1 Grey literature search strategy.

Multimedia Appendix 2 Coding scheme.

Multimedia Appendix 3 Results summary table.

## Abbreviations

EHR: electronic health record
GSSO: gender, sex, and sexual orientation
HCV: hepatitis C virus
SGM: sexual and gender minority
