# Supplementary material for: Culturally Competent Gender, Sex, and Sexual Orientation Information Practices and Electronic Health Records: Rapid Review
Source: JMIR Med Inform. 2021 Feb 11;9(2):e25467. doi: 10.2196/25467 (PMC7906831; doi:10.2196/25467)
Supplement: Multimedia Appendix 1 [file medinform_v9i2e25467_app1.docx]

Appendix 1 - Grey Literature Search Strategy.

**Date Range:** 2015-2020

**Source 1: Medline**

Search String: (sexual orientation or gender identity or sexual minorit* or gender minoriti* or "sex assigned at birth" or MH "Gender Identity+") AND ( electronic health record* or electronic medical record* or electronic patient record*) AND (data or classification or cod* or mining or standard*)

**Source 2: Google**

Search String: (sex OR gender) AND ((electronic health record OR EHR) OR (electronic medical record OR EMR) AND (LGBT OR SGM OR TGNC OR DIVERSE)) filetype:pdf

**Source 3: References sections search**

**Source 4: International working group of GSSO and EHR experts**

| **Included:**   - Grey literature - English language - Within publication range - Focused on definition, collection, use and sharing of GSSO information, not just WHY it should be collected - Not a duplicate | **Excluded:**   - Peer-reviewed journal articles - Other language than English - Outside of publication range - Focused on WHY GSSO data should be collected instead of HOW it should be collected - No reference to EHRs |
| --- | --- |
