# Supplementary material for: Culturally Competent Gender, Sex, and Sexual Orientation Information Practices and Electronic Health Records: Rapid Review
Source: JMIR Med Inform. 2021 Feb 11;9(2):e25467. doi: 10.2196/25467 (PMC7906831; doi:10.2196/25467)
Supplement: Multimedia Appendix 2 [file medinform_v9i2e25467_app2.docx]

Appendix 2 – Coding Scheme.

**Code 1.0 – Review Questions and Objectives**

1.1 Best practices ­for sex and gender information practices in EHR systems

1.2 Gaps, issues, and challenges in sex and gender information practices in EHR systems

1.3 Definitions for sex and gender information practices in EHR systems

1.4 Modernization efforts for sex and gender information practices in EHR systems

1.5 Action plan to modernize sex and gender information practices in EHR systems

1.6 Rules related to lab testing and services

**Code 2.0 - Focus Areas**

2.1 Policy - what is in place to guide definition, collection and use of SG info in EHRs (e.g. Principles, legislation, strategy, directives, regulations, guidelines, privacy)

2.2 Practice - what should be done to define SG info in EHRs (e.g. Current practices, emerging practices and best practice guidance)

2.3 Research - what has been reported in studies that focus on SG info in EHRs

2.4 Community - what do consumers do, feel and want with SG in EHRs –peoples’ voices, especially from LGBT community

2.5 Implementation - what do we know about efforts to put SG info in EHRs

**Code 3.0 Sex and Gender Information**

3.1 Binary, one step question - sex and gender is reported as single field in binary M/F

3.2 Two-step questions (i.e. sex and gender separation)

3.3 Gender identity

3.4 Gender expression/Lived Gender

3.5 Administrative

- terms may also include administrative gender, legal gender, gender ID, legal sex;

- may also include reference to billing, insurance, passports, driver's license

3.6 Clinical sex

3.7 Sex assigned at birth

3.8 Pronouns and name

3.9 Sexual orientation

**Code 4.0 Electronic Health Record System**

4.1 Collection of SG info into EHR (Intake -registration, patient-entered, HCP-entered)

4.2 Use of SG info from EHR (clinical, research, administrative)

4.3 Sharing of SG info between EHRs (interoperability, privacy, etc.)

4.4 Standards, categories, ontology, technology infrastructure

4.5 Processes, information flow and workflow

4.6 Governance of EHR systems

**Code 5.0 Equity**

5.1 Patient rights (e.g. safe environment and privacy)

5.2 Cultural competency

5.3 Guidance, training and education for providers

5.4 Experiences (e.g. stigma and discrimination, misgendering, unwanted disclosure)

5.5 Treatment and screenings and access to care (e.g. mislabelled samples, lack of screening, tests, diagnosis)

5.6 Health outcomes (e.g. reporting on mental health, substance user)

5.7 SG info for clinical care (may include hormone therapy, transitioning, organ inventory)

**Code 6.0 Additional considerations**
