# Supplementary material for: Culturally Competent Gender, Sex, and Sexual Orientation Information Practices and Electronic Health Records: Rapid Review
Source: JMIR Med Inform. 2021 Feb 11;9(2):e25467. doi: 10.2196/25467 (PMC7906831; doi:10.2196/25467)
Supplement: Multimedia Appendix 3 [file medinform_v9i2e25467_app3.docx]

Appendix 3 – Results Summary Table.

| **Title** | **Results/Key Recommendations** |
| --- | --- |
| Committee Report: The Health of LGBTQIA2 Communities in Canada. | Include sections on gender and sexual diversity health needs and inequities, content for training programs, improvements to data collection practices to get more complete picture of the health of SGMs, and include SGMs in all related decision-making. |
| Sexual Orientation, Gender Identity, & Gender Expression (SOGIE) Safer Places Toolkit. | Use language that is used by patients. Language and terms are evolving and fluid. Avoid outdated and offensive terms and phrases. Make care environments explicitly inclusive of SGMs through images, signages and bathroom policies. Ensure confidentiality of GSSO data to patients. |
| Gender-affirming Care for Trans, Two-Spirit, and Gender Diverse Patients in BC: A Primary Care Toolkit. | Respect patients’ self-determination for gender, name, and pronouns.  Be aware of social, medical, and surgical options for transition, and know that all trans folks seek medical gender affirmation. |
| Collecting Sexual Orientation and Gender Identity Data in Electronic Health Records. | Use of two-step question to gather GSSO data, first gender identity and then sex assigned at birth; strongly recommend gathering names used and pronouns on registration forms.  Train all staff on effective communication with LGBT people. Health centers need to work with EHR vendors to create solutions for GSSO data collection, storage, and use. |
| Ready, Set, Go! Guidelines and Tips for Collecting Patient Data on Sexual Orientation and Gender Identity. | Bring the health information technology team early into the process. Train in small groups to make staff comfortable. Have passionate champions to lead the change. Adapt programs to fit the culture of particular health centers. |
| Policy Focus: How to gather data on sexual orientation and gender identity in clinical settings. | Questions about GSSO data should be included in demographic section of intake forms. If not collected at intake, GSSO data should be gathered during the provider visit. |
| Guidelines for the primary and gender-affirming care of transgender and gender nonbinary people. 2nd ed. | An ideal EHR will allow chosen names and pronouns to be displayed for all users in all views. Use two-question to gather gender identity and sex at birth. |
| Guide to Improving Patient Safety in Primary Care Settings by Engaging Patients and Families: Environmental Scan Report. | Interventions need to be adapted to address specific population groups’ needs and circumstances.  Must develop culturally sensitive and appropriate approaches to reaching patients at all literacy levels will be considered. |
| Care Considerations for Inclusion of Gender Diversity within Medical Laboratory Services Presentation. | Information systems should be modified to be inclusive of trans, gender-diverse and nonbinary individuals. The physical environment of labs should be made to be inclusive of SGMs  Avoid terms and phrases that are offensive and outdated. |
| Best Practices: Electronic. Medical Record Data Collection on Sexual Orientation and Gender Identity. | The EHR should include spaces for a patient’s: sexual orientation, gender identity and sex  assigned at birth as a two-part question, chosen name, legal name, and gender pronouns.  The most inclusive practice is to simply leave blanks for the patient to fill in with their identity descriptors. |
| Laboratory Considerations for Care of Gender Diverse Patients. | Make both binary sex references ranges available to clinicians.  Have organization wide standards on how to treat gender X individuals. Update standard operating procedures accordingly. |
| Care Considerations for Inclusion of Gender Diversity within Medical Laboratory Services. | Information systems should be modified to be inclusive of trans, gender-diverse and nonbinary individuals; physical environments should be made to be inclusive of SGMs. Avoid terms and phrases that are offensive and outdated. |
| Exploring the delivery and implementation of primary care services for transgender individuals: An Ontario case study. | EHRs can be a barrier for trans patients accessing healthcare.  Include transgender male, transgender female and gender non-conforming on census forms and data collection.  Remove gender markers from health insurance cards within Canada. |
| BCCDC COVID-19 Language Guidelines for Inclusive Language and Digital Content. | Use inclusive and equitable terms that are relevant to your audience (e.g. use ‘people’, ‘everyone’, or ‘folks’ instead of’ men and women’, ‘you guys’ or ‘guys).  When introducing yourself, include your name and pronouns.  ‘Trans’ is an adjective and should be used as such (e.g. trans man, trans woman). |
| Updates on Terminology of Sexual Orientation and Gender Identity Survey Measures. | Need for future research on the terminology and ordering of response options for GSSO questions. GSSO terminology is fluid and continues to evolve over time. Some SGMs reject the use of labels for sexual orientation and gender identity.  There are many more identities than those listed in questions on GSSO. |
| Standard for sex and gender variables. | Mandatory elements include Male, Female, and Other, please specify. Only one response is permitted. |
| Current Measures of Sexual Orientation and Gender Identity in Federal Surveys working paper. | Need for expanded codes to measure inequity, proper terminology with definitions and rationale. |
| Economic Commission for Europe Conference of Economic Statisticians, 67th Plenary Session Report: In-depth review of measuring gender identity. | Question about gender must include a third option outside of the binary. The benefits of data collection must outweigh the intrusion of privacy. Privacy concerning transgender status is of paramount importance. |
| In Pursuit of Equity: Defining Stratifier for Measuring Health Inequality—A Focus on Age, Sex, Gender, Income, Education and Geographic Location. | Secondary measures (analytics) national equity stratifiers document that outlines data measures for inclusion of SGM including inclusion of Intersex as a sex at birth value, use of two-step question to gather both sex at birth and gender identity, use of appropriate terminology, differences between gender identity (felt gender) and gender expression (lived gender) concepts. |
| Measuring Health Equity: Demographic Data Collection and Use in Toronto Central LHIN Hospitals and Community Health Centres. | Move from ‘one size fits all’ to a personalized medicine. Foster a respectful and caring environment. Educate staff on the potentials of GSSO data to improve care. |
| Canadians can now identify as gender “X” on their passports. | Canadians can change their gender designation to ‘X’ instead of one of the binary gender markers, ‘M’ or ‘F’. |
| Choose or update the gender identifier on your passport or travel document. | The three options for sex designation are F for Female, M for Male, and X for another gender for Canadian passports and travel documents. |
| Gender on health cards and drivers’ licenses. | Sex designation is no longer displayed on the Ontario health card. Driver’s license holder can have ‘X’ displayed to indicate they do not exclusively identify as male or female. |
| Change your personal information. | B.C. residents can change their gender designation on government issued identification without the requirement for surgery. |
| Change of Sex Designation. | Three options for sex designation in Manitoba are Male (M), Female (F), and Gender Diverse (X). |
| An Overview of Alberta’s Electronic Health Record Information System. | Ultimate goal is to have one EHR for each patient that contains all their health data and interactions with the eHealth ecosystem. |
